# Addendum: Werner, D.; et al. Early Immunocastration of Pigs: From Farming to Meat Quality. *Animals* 2021, *11*, 298

**DOI:** 10.3390/ani11040996

**Published:** 2021-04-02

**Authors:** Daniela Werner, Lisa Baldinger, Ralf Bussemas, Sinje Büttner, Friedrich Weißmann, Marco Ciulu, Johanna Mörlein, Daniel Mörlein

**Affiliations:** 1 Institute of Organic Farming, Johann Heinrich von Thuenen Institute, Trenthorst 32, 23847 Westerau, Germany; daniela.werner@thuenen.de (D.W.); lisa.baldinger@thuenen.de (L.B.); ralf.bussemas@thuenen.de (R.B.); SinjeBuettner@gmx.net (S.B.); friedrich.weissmann@gmx.de (F.W.); 2 Department of Animal Sciences, University of Goettingen, Kellnerweg 6, 37077 Göttingen, Germany; marco.ciulu@uni-goettingen.de (M.C.); johanna.moerlein@uni-goettingen.de (J.M.)

The authors wish to add the following to this paper [1]:

*2.1. Experimental Design and Animals*

A total of 55 boars were objected to an early immunization scheme (EARLY) and 54 to the standard immunization scheme (CONTROL) via two consecutive injections (2 mL per injection) of GnRH-Analogon Improvac^®^ (Zoetis, Berlin, Germany).
